# Small Molecules in the Treatment of Squamous Cell Carcinomas: Focus on Indirubins

**DOI:** 10.3390/cancers13081770

**Published:** 2021-04-07

**Authors:** Mirijam Schäfer, Marie Luise Semmler, Thoralf Bernhardt, Tobias Fischer, Vinodh Kakkassery, Robert Ramer, Martin Hein, Sander Bekeschus, Peter Langer, Burkhard Hinz, Steffen Emmert, Lars Boeckmann

**Affiliations:** 1 Clinic and Policlinic for Dermatology and Venereology, University Medical Center Rostock, 18057 Rostock, Germany; mirijam.schaefer@med.uni-rostock.de (M.S.); luise.semmler@med.uni-rostock.de (M.L.S.); thoralf.bernhardt@med.uni-rostock.de (T.B.); tobias.fischer@med.uni-rostock.de (T.F.); steffen.emmert@med.uni-rostock.de (S.E.); 2 Department of Ophthalmology, University of Lübeck, 23562 Lübeck, Germany; vinodh.kakkassery@uni-luebeck.de; 3 Institute for Pharmacology and Toxicology, University Medical Center Rostock, 18057 Rostock, Germany; robert.ramer@med.uni-rostock.de (R.R.); burkhard.hinz@med.uni-rostock.de (B.H.); 4 Institute for Chemistry, University Rostock, 18059 Rostock, Germany; martin.hein@uni-rostock.de (M.H.); peter.langer@uni-rostock.de (P.L.); 5 ZIK *Plasmatis*, Leibniz-Institute for Plasma Science and Technology (INP), 17489 Greifswald, Germany; sander.bekeschus@inp-greifswald.de

**Keywords:** indirubins, small molecules, squamous cell carcinoma

## Abstract

**Simple Summary:**

In this review, the genetic landscape of squamous cell carcinoma is related to the potential targets of indirubin-based small molecules in cancer therapy. Being a component of traditional Chinese medicine, indirubins are used to treat chronic or inflammatory diseases, and have received increasing attention in cancer treatment due to their proapoptotic and antiproliferative activity. Frequent genetic alterations of squamous cell carcinomas are summarized, and it is discussed how these may render tumors susceptible to indirubin-based small molecule inhibitors.

**Abstract:**

Skin cancers are the most common malignancies in the world. Among the most frequent skin cancer entities, squamous cell carcinoma (SCC) ranks second (~20%) after basal cell carcinoma (~77%). In early stages, a complete surgical removal of the affected tissue is carried out as standard therapy. To treat advanced and metastatic cancers, targeted therapies with small molecule inhibitors are gaining increasing attention. Small molecules are a heterogeneous group of protein regulators, which are produced by chemical synthesis or fermentation. The majority of them belong to the group of receptor tyrosine kinase inhibitors (RTKIs), which specifically bind to certain RTKs and directly influence the respective signaling pathway. Knowledge of characteristic molecular alterations in certain cancer entities, such as SCC, can help identify tumor-specific substances for targeted therapies. Most frequently, altered genes in SCC include *TP53*, *NOTCH*, *EGFR*, and *CCND1*. For example, the gene *CCND1*, which codes for cyclin D1 protein, is upregulated in nearly half of SCC cases and promotes proliferation of affected cells. A treatment with the small molecule 5′-nitroindirubin-monoxime (INO) leads to inhibition of cyclin D1 and thus inhibition of proliferation. As a component of Danggui Longhui Wan, a traditional Chinese medicine, indirubins are used to treat chronic diseases and have been shown to inhibit inflammatory reactions. Indirubins are pharmacologically relevant small molecules with proapoptotic and antiproliferative activity. In this review, we discuss the current literature on indirubin-based small molecules in cancer treatment. A special focus is on the molecular biology of squamous cell carcinomas, their alterations, and how these are rendered susceptible to indirubin-based small molecule inhibitors. The potential molecular mechanisms of the efficacy of indirubins in killing SCC cells will be discussed as well.

## 1. Introduction

Skin cancers are the most common malignancies in the world. Among the most frequent skin cancer entities, squamous cell carcinoma (SCC) ranks second (~20%) after basal cell carcinoma (~77%) [1]. While basal cell carcinomas rarely metastasize (0.0028–0.55% [2]), SCC spreads in about 5% [3] of the cases. Standard therapy of early-stage SCC (stadium I and II) [4,5] is the complete surgical removal of the tumor, frequently accompanied by removing the sentinel lymph nodes to prevent metastasis. If surgical removal is not possible, there are different therapeutic options, such as cryotherapy or local immunotherapy, chemotherapy or radiation therapy [5,6]. Patients with locally advanced SCC (stage III and IVa/b) are in most cases treated sequentially with platinum-based chemotherapy with or without induction chemotherapy [4,5]. Due to frequent side effects, caused by toxicity of some chemotherapeutic agents to healthy cells [7,8] or the risk of infection during surgery, targeted therapies are gaining attention. For example, receptor tyrosine kinase (RTK) inhibitors are used to target tumor cells specifically. Lapatinib (Tykerb^®^, GlaxoSmithKline, London-Brentford, UK) is such an inhibitor targeting the receptor tyrosine kinase and EGFR-member ErbB2 to treat breast cancer (BC). In the ALTERNATIVE study, Lapatinib was tested in combination with the monoclonal antibody Trastuzumab (Herceptin^®^, Hoffmann-La Roche, Basel, Switzerland) and an aromatase inhibitor (AI) versus Trastuzumab + AI [9]. The progression-free survival (PFS) increased in the Lapatinib combination to 11 months versus 5.6 months in the Trastuzumab + AI group (HR 0.62 (95%Cl 0.45–0.88)) [9]. A combination of Lapatinib with AI alone resulted in a PFS of 8.3 months (HR 0.85 (95%Cl 0.62–1.17)) [9]. Because EGFR is overexpressed in a lot of SCC cases, there are even studies using short-term Lapatinib for the treatment of actinic keratosis (AK) and cutaneous SCC [10]. The treatment resulted in tumor regression in 2 out of 8 patients and a reduced AK in 7 out of 8 patients [10]. Frequently altered signaling pathways in cancers are involved in proliferation, migration, invasion and metastasis, angiogenesis, and apoptosis [11]. One way to target these pathways for therapeutic purposes is through the use of these small molecule inhibitors.

As a component of Danggui Longhui Wan, a traditional Chinese medicine, indirubins are used to treat chronic diseases [12] and have been shown to inhibit inflammatory reactions [13,14]. Indirubins are pharmacologically relevant small molecules with pro-apoptotic and antiproliferative activity. The first molecules based on indirubins appeared in literature in the 1980s [15,16]. The number of publications about indirubins in cancer therapy increased slightly until the 1990s, stagnated for almost ten years, and received more attention in cancer research since the 2000s. Indirubins have been shown to inhibit DNA synthesis, protein kinases [17,18,19], and cyclin-dependent kinases [19]. Therefore, they intervene directly with signaling pathways frequently altered in cancer cells and, thus, have the potential to prevent tumor cell proliferation and augment cytotoxicity.

In this review, we discuss the current literature on indirubin-based small molecules in cancer treatment. A particular focus is on the molecular biology of squamous cell carcinomas, their alterations, and how this may render these tumors particularly susceptible to indirubin-based small molecule inhibitors. Potential molecular mechanisms of indirubins-induced toxicity in cancer cells will be discussed.

## 2. Small Molecules

The term small molecule is generally imprecisely defined, so the term is used in many areas. Generally, sources state that small molecules are a heterogeneous group of active molecules with a maximum weight of 900 g/mol [20]. They are produced by chemical synthesis or fermentation [21] and characterized by various biological functions. These include signal transduction, as a medicinal product or pesticide. About 2% of small molecules are so small that they can cross the blood–brain barrier, allowing indirubin-based drugs to be effective against insomnia, depression, or schizophrenia [22]. Small molecular compounds are also gaining in importance for targeted tumor treatments [23], which will be discussed in more detail below.

## 3. Mode of Action of Small Molecules and Potential Targets in Cancer

Depending on the class of substance, small molecules have the property of inhibiting or activating specific intracellular signaling pathways [24,25,26], leading to reactivation of apoptosis mechanisms in cancer cells [27]. 58 human RTKs are known to play a key role in oncogenesis [28] and many small molecules target RTKs. RTK inhibitors are divided into five groups: type I inhibitors are noncovalent ATP-competitive inhibitors that link to active conformation [29]; type II inhibitors are nonselective by remaining in their inactive conformation and binding next to the ATP-binding site of inactive kinases [29]; in contrast, type III inhibitors are highly selective binding an allosteric site, remote from ATP site [29,30]; type IV RTKIs reversibly bind to the substrate-binding site of the kinase; type V inhibitors bind covalently and irreversibly to the active center of the kinase [29]. 

Meanwhile several small molecule RTKIs are approved as monotherapy or in combination therapies and provide favorable risk-to-benefit profiles compared to other therapies, such as cytostatic or radiation therapies [25,26]. Following the identification of the activating BRAF-V600 mutation in melanoma, the BRAF targeting small molecule Vemurafenib was approved for cancer treatment by the Federal Drug Administration (FDA) in 2011. A phase 3, randomized open-labeled study named BRIM-3 assessed the effects of Vemurafenib on patients with BRAF-V600E- and BRAF-V600K-positive melanoma in comparison to the cytostatic Dacarbazine [31]. This study revealed a median overall survival of 13.6 months (95%Cl 12.5–15.2) in the Vemurafenib group versus 9.7 months (95%Cl 7.9–12.8) in the Dacarbazine group [31]. The median progression-free survival was 6.9 months (95%Cl 6.1–7.0) in the Vemurafenib group versus 1.6 months (95%Cl 1.6–2.1) in the Dacarbazine group [31]. IMspire150 was another phase 3, randomized open-labeled study dealing with BRAF-V600-positive melanoma patients treated with Vemurafenib and the mitogen activated protein kinase (MEK)-inhibitor Cobimitinib with and without addition of the monoclonal programmed cell death ligand 1 (PD-L1) antibody Atezolizumab [32]. The combination of both inhibitors with the antibody therapy improved progression-free survival from 10.6 months to 15.1 months (Hazard ratio 0.78; 95% CI 0.63–0.97; *p* = 0.025) [32]. Gutzmer and colleagues interpreted the addition of Atezolizumab to targeted therapy with Vemurafenib and Cobimetinib as “safe and tolerable and significantly increased progression-free survival in patients with BRAFV600 mutation-positive advanced melanoma” [32]. These examples demonstrate that identifying tumor-specific mutations, and the development of small molecules that specifically target these alterations to restore the disturbed signaling pathway, is a promising strategy for precision cancer therapy. 

Targeting genetic alterations is one of four tracks (genetics track) for targeted cancer therapies described by Benson and colleagues (Table 1) [33]. Gene mutations, and stable changes in expression across multiple cell divisions in cancer cells, are used to target and eliminate exactly these cells. The synergy track aims to restore apoptotic signaling pathways or induce synthetic lethality by targeting a mechanism in the cell that became essential, due to defects in another mechanism. An example for inducing synthetic lethality using small molecules is the poly(ADP-ribose)-polymerase (PARP) inhibitor DDHCB for the treatment of patients with *breast cancer gene* (*BRCA*) mutations [34]. BRCA plays a role in DNA double-strand break (DSB) repair through homologous recombination (HR). Inhibition of PARP (plays a role in base excision repair (BER)) leads to an increase of DSBs, which subsequently overwhelms the repair capacity of the homologous recombination pathway [35]. Consequently DSBs can only be repaired by error-prone non-homologous end joining (NHEJ) [35]. Hence, the defect in HR combined with BER inhibition leads to errors in DSB repair and subsequently to the induction of apoptosis. According to Benson and colleagues [33] (Table 1), the third track is the lineage track. This track aims at dependencies of cancer cells on tissue and cell type-specific survival factors, e.g., the microphthalmia-associated transcription factor (MITF)-inhibitor ML329, described by Faloon and colleagues [36] (Table 1). The last track Benson and colleagues describe is the host track. Inhibition or disturbance of the tumor environment leads to growth inhibition or starvation of the cancer cells. For example, the vascular endothelial growth factor receptor (VEGFR)- and rapidly accelerated fibrosarcoma-1 (Raf-1)-inhibitor Sorafenib inhibits angiogenesis in renal cancer cells and has been shown to partially stabilize the disease or shrink the tumor [37].

Comprehensive knowledge of impaired functions in specific tumors crucial for carcinogenesis allows the development of drugs that specifically target these functions in different tracks (Table 1). Hence, genetic analyses of different cancer entities provide the basis for specific targeted therapies using small molecules. 

**Table 1:** Different tracks for targeted therapies using small molecules according to Benson et al. [33].

Target	Background	Example
Genetics track	Stable changes in DNA (gene mutation and expression) across multiple cell divisions	BRAF-MEK-Inhibitors e.g., Vemurafenib (PLX4032) [31,37,38]
Synergy track	Restoring apoptotic signaling pathways or inducing synthetic lethality by targeting mechanism that became essential due to defects in another mechanism	PARP-Inhibitors for patients with *BRCA* mutation e.g., DDHCB [34]
Lineage track	Inhibition of tissue and cell type-specific survival factors	MITF-Inhibitors e.g., ML329 [36]
Host track	Inhibition/disturbance of the tumor environment (inhibition of angiogenesis)	VEGFR-Inhibitors e.g., Sorafenib (BAY 43-9006) [37]

## 4. Molecular Biology of Squamous Cell Carcinoma

In this review, we focus in particular on genetic alteration frequently observed in squamous cell carcinomas of the skin (SCC). Most frequently, mutated or misregulated in SCC are the tumor suppressor protein gene *TP53*, downregulated in 42–90% of SCC cases [39,40,41,42] and *NOTCH*, downregulated in 22–86% of cases [39,43,44,45,46] (Table 2). The protein p53 normally ensures a cell cycle arrest by inhibiting the cyclin D/CDK4/6- and the cyclin E/CDK2-complex if the cell is damaged and initiates apoptosis by activating *b-cell lymphoma-2* (*Bcl-2)*-genes [47]. A mutation or loss of function of *TP53* may lead to uncontrolled cell growth and cancer. NOTCH is a transmembrane protein that forms a complex with other proteins leading to the induction of NOTCH-response genes such as *hairy and enhancer of slit-related* genes (*HESR*) [48], *cellular myelocytomatosis* (*c-Myc*) [49,50], *cyclin D1* (*CCND1*) [51], *cyclin D3* (*CCND3*) [52], *cyclin-dependent kinase 5* (*CDK5*) [50], the cyclin-dependent kinase (CDK)-inhibitor *p21* [53], the *zinc finger protein family snail* gene (*SNAI1*) [54] and the *platelet-derived growth factor receptor β* gene (*PDGFRβ*) [55]. These NOTCH-response genes are involved in proliferation, cell differentiation and angiogenesis. The negative regulator of *NOTCH* and proliferation factor EGFR is overexpressed in 43–95% of SCC tumors [56,57,58,59,60,61] (Table 2). EGFR is a transmembrane receptor with intrinsic tyrosine kinase activity. It is activated by dimerization of EGF and TGFα, which activates signaling molecules such as signal transducers and activators of transcription (STAT), protein kinases B (Akt/PKB), and MEK and leads to the stimulation of cell growth and the prevention of apoptosis [62]. The gene *CCND1*, overexpressed in 30–50% of SCC cases [63,64,65], encodes the cyclin D1 protein. Cyclins act as regulators for cyclin-dependent kinases (CDKs). Cyclin D1, in particular, forms a complex with CDK4 or CDK6 and acts as their regulatory subunit [66,67,68]. The complex monophosphorylates and activates the retinoblastoma protein (pRb) in DNA damage response [69]. The monophosphorylated pRb then binds the transcription factor E2F, which leads to a cell cycle arrest in the G1-phase [69]. The activation of the cyclin E/CDK2 complex at the late G1 restriction point hyper phosphorylates and inactivates pRb, E2F is split off, and the cell cycle continues in the S-phase. 

A loss of function of *CDKN2A* (*cyclin-dependent kinase inhibitor 2A*), which codes for two proteins, is frequently observed in SCC (28%; Table 2) [40,70,71]. One protein *CDKN2A* encodes for is the INK4 member p16. This tumor suppressor regulates the cell cycle by inhibiting CDK4 and CDK6, thereby preventing phosphorylation and activation of pRb. Inactive pRb is not bound to E2F and the cell cycle continues [69]. A feedback loop is generated in which the expression of p16 is controlled by the retinoblastoma proteins [72,73]: the pRb-E2F complex inhibits the expression of p16, less p16 inhibits the cyclin D1/CKD4/6 complex and more active pRb binds E2F. The p16/Rb signaling pathway collaborates with the mitogenic signaling cascade to induce reactive oxygen species, which activate the protein kinase C delta and lead to an irreversible cell cycle stop [74,75]. The other protein encoded by the *CDKN2A* gene is p14ARF. This protein activates the tumor suppressor p53, induces cell cycle arrest in the G2 phase and subsequent apoptosis [76]. In addition, p14ARF is said to downregulate E2F-dependent transcription and would therefore also play a role in controlling the G1/S transition [77]. The gene for transforming growth factor-beta receptors (*TGFBR*) encodes for serine/threonine kinase receptors, which are involved in cell differentiation [78] and apoptosis [79]. A loss of TGFBR, observed in 43% of SCC cases [46] (Table 2), leads to increased proliferation of the cells [80]. The next gene is *HRAS* (*Harvey rat sarcoma*), which encodes for the GTPase HRAS and is upregulated in 6–38% of SCC cases (Table 2). It plays a role in cell growth, division and survival by regulating the RAF/MAPK/ERK [81] and P13K/Akt pathway [82]. *Kinetochore localized astrin/SPAG5 binding protein* gene (*KNSTRN*) encodes for a protein responsible for modulation of anaphase onset and chromosome segregation during mitosis [40]. In SCC patients, *KNSTRN* is mutated in 17–19% of cases and is associated with controlling chromosomal activity in normal and cancerous cells [40,83]. Lee and colleagues (2014) examined recurrent point mutations in the *KNSTRN* gene in cutaneous squamous cell carcinomas by sequencing, and sequencing libraries in vitro and in vivo. They show mutant *KNSTRN* disrupts chromatid cohesion required for faithful chromosome segregation, driving cells toward aneuploidy and leading to tumor development [40].

Table 2 summarizes the discussed, most frequently observed genetic alterations in squamous cell carcinomas. The growing understanding of such genetic alterations underlying carcinogenesis, and the further development of various methods for small molecule synthesis, allows the generation of an ever-broader spectrum of potentially active molecules. Once the defective genes are known, existing and novel small molecules can be screened for molecules that target these altered pathways and selectively eliminate the cancer cells.

**Table 2:** Frequently altered genes in squamous cell carcinomas.

Gene	Altered in SCC	Reference
*TP53*	42–90% ↓ Mutation	Nakazawa et al. (1994) [42] Giglia-Mari et al. (2003) [41] Lee et al. (2014) [40] Inman et al. (2018) [39]
*NOTCH*	22–86% ↓	Stransky et al. (2011) [43] South et al. (2012) [44] South et al. (2014) [45] Cammareri et al. (2016) [46] Inman et al. (2018) [39]
*EGFR*	43–95% ↑ Overexpression	Rodeck et al. (1997) [56] Ang et al. (2002) [58] Kaliankrishna et al. (2006) [61] Fogarty et al. (2007) [59] Kolev et al. (2008) [60] Uribe et al. (2011) [57]
*CCND1*	30–50% ↑ Overexpression	Bartkova et al. (1995) [63] Izzo et al. (1998) [64] Ikeguchi et al. (2001) [65]
*TGFBR*	43% ↓ Mutation/Loss	Cammareri et al. (2016) [46]
*HRAS*	6–38% ↑	Bamford et al. (2004) [84] Durinck et al. (2011) [85] South et al. (2014) [45] Lee et al. (2014) [40] Cammareri et al. (2016) [46] Inman et al. (2018) [39]
*CDKN2A* (*p16INK4a*)	28% ↓ Mutation/Loss	Brown et al. (2004) [71] Bäckvall et al. (2005) [70] Lee et al. (2014) [40]
*KNSTRN*	17–19% ↓ Mutation	Lee et al. (2014) [40]

↑ Activation   ↓ Inactivation.

## 5. Small Molecules Based on Indirubins in the Treatment of SCC

**Figure 1:**
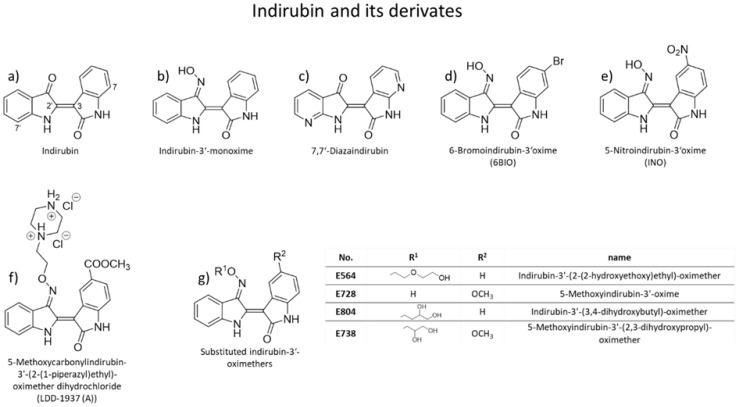
Indirubine and its derivates. (**a**) Indirubin; (**b**) Indirubin-3′-monoxime; (**c**) 7,7′-Diazaindirubin; (**d**) 6-Bromoindirbun-3′-oxime (6BIO); (**e**) 5-Nitroindirubin-3′oxime (INO); (**f**) 5-Methoxycarbonylindirubin-3’-(2-(1-piperazyl)ethyl)-oximether dihydrochloride (LDD-1937 (A)); (**g**) substituted indirubin-3′-oximethers: Indirubin-3′-(2-(2-hydroxyethoxy)ethyl)-oximether (E564), 5-Methoxyindirubin-3′-oxime (E728), Indirubin-3′-(3,4-dihydroxybutyl)-oximether (E804), 5-Methoxyindirubin-3′-(2,3-dihydroxypropyl)-oximether (E738).

For some years now, indirubins have been a promising basic structure for synthesizing of new small molecules for cancer treatment. As a component of Danggui Longhui Wan, a traditional Chinese medicine, indirubins are used to treat chronic diseases [12] and have been shown to inhibit inflammatory reactions [13,14]. With regard to cancer, they have been shown to inhibit DNA synthesis, protein kinases [17,18,19], and cyclin-dependent kinases [19]. This means that they may interfere with frequently disturbed signaling pathways in SCC and thus provide good candidates to stop tumor cell growth. Specifically, this involves intervening in proliferation by inhibiting the dimerization and phosphorylation of the receptor tyrosine kinase c-Met to stop the subsequent signaling pathways P13K/Akt, RAS/MAPK, and STAT [86]. The tyrosine kinase receptor c-Met normally binds with hepatocyte growth factor (HGF) and triggers processes such as embryogenesis, cell growth, cell differentiation, and angiogenesis [87,88]. Yasui and colleagues [89] demonstrated in SCC-cells the induction of the formation of lammellipodia by c-Met signaling, which promotes migration. Ndolo and colleagues [90] tested the indirubin derivate LDD-1937 (5-Methoxycarbonylindirubin-3’-(2-(1-piperazyl)ethyl)-oximether dihydrochloride; Figure 1) in gastric cancer cells SNU-638 (overexpress c-Met), which inhibits migration due to the lack of binding possibility of HGF. The binding possibility of HGF is also associated with the regulation of invasion and metastasis of tumors [91,92,93,94]. Treatment with LDD-1937 also decreased the expression of the Erk1/2, STAT3, STAT5, and Akt (downstream proteins of c-Met) as well as cyclin B1 and CDK2, leading to reduced cell viability, colony formation, and cell cycle arrest in the G2/M phase [90]. Finally, apoptosis was induced, evident by increased cleavage of PARP after treatment with LDD-1937 [90]. 

**Figure 2:**
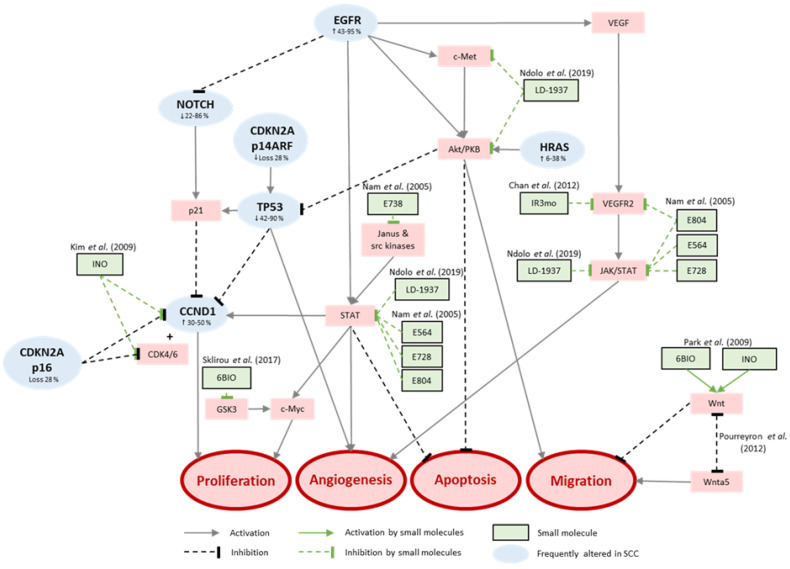
Indirubins interacting with signaling pathways frequently altered in squamous cell carcinoma of the skin.

Another point of attack is the inhibition of EGFR. It is frequently overexpressed in SCC (Table 2 [56,57,59,60]) and involved in proliferation and apoptosis [62]. The inhibition by AG1478 and Cetuximab suppresses migration and invasion in tongue SCC cells SAS and gingival SCC cells CA9-22. It was also shown that the migration of SCC cells is negatively influenced by inhibition of Wnt5a [91,93], which is strongly expressed in nonmelanoma skin cancer [91]. Pourreyron and colleagues showed that Wnt5a is forming active gradients, while canonical Wnt signaling is repressed [91]. The inhibition of Wnt5a and the activation of the canonical Wnt signaling pathway provide further potential targets of an indirubin-based small molecule therapy. Park and colleagues found that the indirubin INO (5-Nitroindirubin-3′-oxime, Figure 1) as well as bromindirubin-3-oxime, could be an activator of Wnt (Figure 2) and the associated canonical β-catenin-mediated signaling path, similar to what has been observed for [95].

Furthermore, treatment of human breast and prostate cancer cells with the indirubin derivates E564 (Indirubin-3′-(2-(2-hydroxyethoxy)ethyl)-oximether, Figure 1), E728 (5-Methoxyindirubin-3′-oxime, Figure 1), and E804 (Indirubin-3′-(3,4-dihydroxybutyl)-oximether, Figure 1) showed STAT3 inhibition leading to decreased growth [96] and induction of apoptosis (Figure 2 [96,97,98]). E738 (5-Methoxyindirubin-3′-(2,3-dihydroxypropyl)-oximether, Figure 1) has been identified as inhibitor of janus and src family kinases, observed in human pancreatic cancer cells (Panc-1, MIA-PC2, BXPC3, AsPC1) [18]. Inhibition of these kinases leads to the inhibition of STAT3 and subsequently reduces proliferation and induces apoptosis [18]. These indirubin derivates have not been tested in SCC cells so far, but since EGFR, which activates STAT3, is frequently overexpressed in SCC (Table 2), targeting STAT3 may provide a promising approach for SCC treatment as well. STAT3 normally induces the expression of downstream targets *BcL-2*, *b-cell lymphoma xL (Bcl-xL)*, *induced myeloid leukemia cell differentiation protein* gene (*Mcl-1*), *c-Myc, surviving,* and *CCND1* [99]. Bcl-2, Bcl-xL, and Mcl-1 are antiapoptotic proteins of the Bcl-2 protein family [100]. Bcl-2 and Bcl-xL control mitochondrial membrane permeability and the release of cytochrome c, which modulates apoptosis [101]. Therefore, inhibition of STAT3 also reduces the level of Bcl-xL. As a consequence of missing Bcl-xL, the mitochondrial membrane potential destabilizes, cytochrome c gets released, and apoptosis is induced (Figure 2 [102,103]). Mcl-1 is an exceptional player of Bcl-2 family. A loss of function of Mcl-1 has the most dramatic impact on cell survival of different cell types [100]. Another downstream target is *c-Myc*. *MYC* is a transcription factor, which affects regulation of most active human genes in the cell without any particular preference [104]. Mutated *MYC* is an oncogene because it can permanently upregulate the expression of certain genes [105,106,107,108,109]. Overexpression of *survivin* in cancer cells is associated with a significantly reduced survival rate of the affected patients [56,59,65], a higher likelihood of recurrence, and a reduced rate of apoptosis in tumor cells. The last downstream target of STAT3 mentioned here is *CCND1*, which is overexpressed in up to 50% of SCC tumors (Table 2 [63,64,65]) and encodes for cyclin D1. It is involved in the transition from G1 phase to S phase of the cell cycle [66,67,68] by binding CDK4/6 and phosphorylating pRb. Kim and colleagues [110] reported that 5′-nitroindirubin-monxime (INO, Figure 1) inhibits the proliferation of human SCC cells (KB cells) by reducing CDK4 and cyclin D1/cyclin D3 levels leading to cell cycle arrest in the G1/S phase and by reducing the activity of cyclin-dependent kinase 2/cyclin B complex, which induces a cell cycle arrest in G2/M phase. Indeed, Akiyama and colleagues [111] showed that STAT3 inhibition by STAT shRNA-4 caused suppression of tumor growth and induction of apoptosis by upregulation of tumor suppressor latexin in highly STAT3-activated SCC-3 cells. 

Like inhibition of STAT3, the inhibition of glycogen synthase kinase 3 (GSK3) by (2′Z, 3′E)-6-bromoindirubin-3′-oxime (6BIO; Figure 1), as shown in Figure 2, also reduces the expression of *MYC* in human embryonic fibroblasts cells [112]. By using 6BIO in combination with doxorubicin (DXT) for treatment of human newborn foreskin (BJ cells) and human lung embryonic fibroblasts (IMR90 cells) Sklirou and colleagues demonstrated a reduced activation of p53 and less γH2AX phosphorylation than by using DXT alone.

Besides their impact on proliferation, migration, and apoptosis, indirubin and its derivates indirubin-3′-monoxime (IR3mo; Figure 1) and E804) also decrease angiogenesis by inhibiting VEGFR2-dependent JAK/STAT3 signaling [13,113]. By preventing VEGFR2 phosphorylation at two phosphorylation sites [39]. It was also shown that the *NOTCH* gene, which is crucial for angiogenesis and mutated in SCCs in 22–86% of cases (Table 2 [39,43,44,45,46]), is activated by c-Met signaling. Consequently, inhibition of c-Met also leads to reduced NOTCH activity, ultimately reducing angiogenesis. Direct inhibition of NOTCH1 by IR3mo has already been demonstrated [114], as well as inhibition of DRAK2 (DAP kinase-related apoptosis-inducing protein kinase 2) [12]. Jung and colleagues identified indirubin-3-monoximes by a high throughput screening campaign, in which 16 potent indirubin-based inhibitors were found to inhibit DRAK2 [12]. It belongs to the superfamily of death-associated protein kinase (DAPK)-family and serves to set the initial threshold for thymic and peripheral T-cell activation and later, to maintain the survival of effector T cells [115,116]. Moreover, it has been shown that ectopic expression of DRAK2 in cell lines induces apoptosis [117,118].

Finally, Cheng and colleagues identified 7,7-Diazaindirubin (Figure 1) as a cause of inhibition of casein kinase 2 in LXFL529L cells (human large cell lung tumor xenograft). It exhibited markedly enhanced growth inhibitory activity in these cells [119]. This substance also displayed antiproliferative activity in the National Cancer Institute (NCI) 60 cell line panel preferentially in certain melanoma and non-small cell lung cancer cells, according to Cheng et al. [119]. The casein kinase 2 has dual functionality, being involved in both cell growth and proliferation as well as apoptosis [120].

The above-described activities of several indirubin based small molecules and their role in inhibiting proliferation, angiogenesis, and/or migration as well as inducing apoptosis combined with knowledge on frequent molecular alterations in SCCs that lead to the activation of proliferation, angiogenesis, and/or migration as well as the inhibition of apoptosis suggest that indirubin derivates are promising candidates for SCC treatment. Hence, further studies assessing the molecular mechanisms of existing indirubin-based small molecules as well as of newly synthesized indirubin derivates are warranted.

## 6. Conclusions

This review summarizes frequently altered genes in SCC and shows how different indirubin derivates directly or indirectly interfere with these genes by inhibiting proteins downstream or upstream of the altered genes. Although some of the effects of indirubins are known, more research is needed to elucidate the molecular mechanisms of action. Knowledge of characteristic molecular alterations in certain cancer entities, such as those shown here for SCC, can help identify tumor-specific substances for targeted therapies. Indirubins show a broad spectrum of activity against SCC and, hence, provide a class of substances with further potential for targeted SCC therapies.

## Data Availability

Not applicable.
